# Bioinformatics Analysis for the Antirheumatic Effects of Huang-Lian-Jie-Du-Tang from a Network Perspective

**DOI:** 10.1155/2013/245357

**Published:** 2013-11-21

**Authors:** Haiyang Fang, Yichuan Wang, Tinghong Yang, Yang Ga, Yi Zhang, Runhui Liu, Weidong Zhang, Jing Zhao

**Affiliations:** ^1^Department of Mathematics, Logistical Engineering University, Chongqing 401311, China; ^2^Tibet Traditional Medical College, Lhasa 850000, China; ^3^The National Medical College, Chengdu University of TCM, Chengdu 610075, China; ^4^Department of Natural Medicinal Chemistry, Second Military Medical University, Shanghai 200433, China

## Abstract

Huang-Lian-Jie-Du-Tang (HLJDT) is a classic TCM formula to clear “heat” and “poison” that exhibits antirheumatic activity. Here we investigated the therapeutic mechanisms of HLJDT at protein network level using bioinformatics approach. It was found that HLJDT shares 5 target proteins with 3 types of anti-RA drugs, and several pathways in immune system and bone formation are significantly regulated by HLJDT's components, suggesting the therapeutic effect of HLJDT on RA. By defining an antirheumatic effect score to quantitatively measure the therapeutic effect, we found that the score of each HLJDT's component is very low, while the whole HLJDT achieves a much higher effect score, suggesting a synergistic effect of HLJDT achieved by its multiple components acting on multiple targets. At last, topological analysis on the RA-associated PPI network was conducted to illustrate key roles of HLJDT's target proteins on this network. Integrating our findings with TCM theory suggests that HLJDT targets on hub nodes and main pathway in the Hot ZENG network, and thus it could be applied as adjuvant treatment for Hot-ZENG-related RA. This study may facilitate our understanding of antirheumatic effect of HLJDT and it may suggest new approach for the study of TCM pharmacology.

## 1. Introduction

Rheumatoid arthritis (RA) is a chronic, systemic inflammatory joint disorder that principally attacks flexible (synovial) joints, leading to the destruction of articular cartilage and fusion of the joints. It can also affect other tissues throughout the body. RA is considered as a systemic autoimmune disease, whose cause and pathogenesis remain largely unknown.

Currently there is no cure for RA. The aim of the treatment is to reduce inflammation, relieve pain, suppress disease activity, prevent joint damage, and slow disease progression, so as to maintain the patient's quality of life and ability to function. Clinical treatments for RA include nonsteroidal anti-inflammatory drugs (NSAIDs), disease modifying antirheumatic drugs (DMARDs), glucocorticoids, and biological response modifiers. Even so, current RA treatment medications are limited by several well-characterized clinical side effects, such as hepatotoxicity [1, 2], gastrointestinal effects [3], and cardiotoxic effects [4]. Therefore, there is a need to explore new or alternative anti-RA agents.

Huang-Lian-Jie-Du-Tang (HLJDT; oren-gedoku-to in Japanese), a classic TCM formula to clear ‘‘heat” and ‘‘poison,” is an aqueous extract of four herbal materials, Rhizoma Coptidis, Radix Scutellariae, Cortex Phellodendri, and Fructus gardeniae. It has been used to treat gastrointestinal disorders, inflammation, liver disease, hypertension, and cerebrovascular disease [5]. Earlier studies have demonstrated that HLJDT possesses antiobesity [6], antitumor [7], neuroprotection [8], and anti-inflammatory activities [9, 10]. A series of experimental studies by one of our laboratories on HLJTD's effects on collagen-induced arthritis in rats suggested that HLJDT exhibits antirheumatic activity [11–13]. On the other hand, many compounds have been identified as active ingredients of HLJDT, including baicalin, baicalein, wogonoside, wogonin, berberine, coptisine, palmatine, jatrorrhizine, crocin, crocetin, chlorogenic acid, and geniposide [14], some of which have been reported to show antirheumatic effects [15–18].

It has been known that complex chronic diseases including RA are usually caused by an unbalanced regulating network resulting from the dysfunctions of multiple genes or their products [19–22]. Meanwhile, as multicomponent and multitarget agent, the therapeutic effectiveness of a TCM formula is believed to be achieved through collectively modulating the molecular network of the body system by its active ingredients [23, 24]. Thus there is a need to study the therapeutic mechanism of TCM formulae on complex diseases from the viewpoint of network-based systems biology [23–28].

In this work, we studied antirheumatic effects of HLJDT as compared to FDA-approved anti-RA drugs from network perspective. We first collected genes associated with RA, proteins inhibited by main active compounds of HLJDT, and targets of FDA-approved anti-RA drugs. Then we study the drug targets in the context of RA-associated pathway and protein networks. HLJDT's targets were mapped onto the drug-target network of FDA-approved anti-RA drugs and the RA pathway in the KEGG database to investigate their potential anti-RA functions. The network-based antirheumatic effect score was defined to quantitatively analyze the antirheumatic effect of HLJDT and compare it with those of FDA-approved anti-RA drugs. Topological analysis on the RA-associated PPI network was conducted to explore the roles that HLJDT's target proteins play on this network.

## 2. Materials and Methods

### 2.1. Data Preparing

#### 2.1.1. RA-Associated Genes

We collected genes associated with RA from three resources as follows.

1. The Online Mendelian Inheritance in Man (OMIM) database [29]: it is a database that catalogues all the known diseases with a genetic component and when possible links them to the relevant genes in the human genome and provides references for further research and tools for genomic analysis of a catalogued gene. We searched the OMIM database with a keyword “rheumatoid arthritis” and found 7 causal genes: CD244, HLA-DR1B, MHC2TA, NFKBIL1, PAD, SLC22A4, and PTPN8.
2. Genetic Association Database (GAD) [30]: it is an archive of human genetic association studies of complex diseases and disorders and includes summary data extracted from published papers in peer-reviewed journals on candidate gene and GWAS studies. We searched the GAD database with a keyword “rheumatoid arthritis” and found 82 genes whose association with RA was shown “Y.” Five of the seven RA causal genes in the OMIM database are also included in the 82 genes collected from the GAD.
3. Kyoto Encyclopedia of Genes and Genomes (KEGG) Pathway Database [31]: this is a collection of online databases dealing with genomes, enzymatic pathways, and biological chemicals. A total of 92 genes appear on the rheumatoid arthritis pathway in the KEGG database. These genes are considered to be associated with RA.

Based on the above three databases, we obtained 163 distinct genes that are associated with RA (see Table S1 in Supplementary Material available online at http://dx.doi.org/10.1155/2013/245357).

#### 2.1.2. FDA Approved Anti-RA Drugs and Their Target Proteins

The data of FDA-approved anti-RA drugs and their targets was downloaded from the DrugBank database [32], which was updated in May 2013. We searched the DrugBank database with a keyword “rheumatoid arthritis” and extracted all of the FDA-approved anti-RA drugs and their corresponding targets (32 drugs and 51 protein targets). Four classes of drugs are used clinically for the treatment of RA. They are nonsteroidal anti-inflammatory drugs (NSAID) such as flurbiprofen, disease-modifying antirheumatic drugs (DMARDs) such as sulfasalazine, glucocorticoids such as cortisone acetate, and biological response modifiers such as etanercept and abatacept. See Supplementary Table S2 for detail.

#### 2.1.3. Target Proteins of HLJDT's Main Ingredients

Based on our pervious study and literature reports, fourteen active components are identified in HLJDT: baicalin, baicalein, wogonoside, wogonin, berberine, magnoflorine, phellodendrine, coptisine, palmatine, jatrorrhizine, crocetin, crocin, chlorogenic acid, and geniposide [10, 14]. Data about target proteins for HLJDT's main compounds was collected from Herbal Ingredients' Targets Database (HIT) [33], a well-known herb ingredient target database (http://lifecenter.sgst.cn/hit/), with a keyword of each ingredient name. According to HIT, 10 ingredients can find the corresponding drug target proteins. They are baicalein, berberine, chlorogenic, coptisine, crocetin, crocin, geniposide, jatrorrhizine, palmatine, and wogonin, in which crocin's only one target could not be found on the PPI network we used. Thus crocin is not included in our network analysis. A total of 91 distinct target proteins of HLJDT were found in the HIT database. The detailed data are shown in Supplementary Table S3.

#### 2.1.4. Protein-Protein Interaction Data

Protein-protein interactions between human proteins were downloaded from the version 9.05 of STRING [34]. STRING includes both physical and functional interactions integrated from numerous sources, including experimental repositories, computational prediction methods, and public text collections. It uses a scoring system to weigh the evidence of each interaction. The interaction scores were normalized to the interval [0, 1]. We first extracted interactions weighted at least 0.9 to construct a protein-protein interaction network with high confidence. Then we checked if the genes we studied, that is, RA-associated genes, FDA-approved anti-RA drugs' target proteins, and target proteins of HLJDT's main ingredients, are included in this network. For those genes missing in this network but appearing in the STRING database, we added their interactions with the highest weights which are less than 0.9. In this way, we constructed a weighted PPI network with 9289 nodes and 57179 edges.

### 2.2. Construction of Drug-Target Network

A drug-target network is defined as a bipartite network for the drug-target associations consisting of two disjoint sets of nodes [35]. One set of nodes corresponds to all drugs under consideration, and the other set corresponds to all the proteins targeted by drugs in the study set. A protein node and a drug node are linked if the protein is targeted by that specific drug according to the DrugBank information.

### 2.3. Pathway Enrichment Analysis

We used pathway enrichment analysis [36] to determine whether a pathway is significantly regulated by HLJDT. Hypergeometric cumulative distribution was applied to quantitatively measure whether a pathway is more enriched with HLJDT's targets than would be expected by chance [37]. Generally, if we randomly draw *n* samples from a finite set, the probability of getting *i* samples with the desired feature by chance obeys hypergeometric distribution as

(1) $$\begin{array}{c} f\left(i\right)=\frac{\left(\begin{array}{c} K\\ i \end{array}\right)\left(\begin{array}{c} N-K\\ n-i \end{array}\right)}{\left(\begin{array}{c} N\\ n \end{array}\right)}, \end{array}$$

where *N* is the size of the set and *K* is the number of items with the desired feature in the set. Then the probability of getting at least *k* samples with the desired feature by chance can be represented by hypergeometric cumulative distribution defined as *P* value:

(2) $$\begin{array}{c} P=1-\sum_{i=0}^{k-1}f\left(i\right)=1-\sum_{i=0}^{k-1}\frac{\left(\begin{array}{c} k\\ i \end{array}\right)\left(\begin{array}{c} N-K\\ n-i \end{array}\right)}{\left(\begin{array}{c} N\\ n \end{array}\right)}. \end{array}$$

Given significance level *α*, a *P* value smaller than *α* demonstrates low probability that the items with the desired feature are chosen by chance. In our case, if all pathways under study include *N* distinct genes, in which *K* genes are HLJDT's targets, for a pathway with *n* genes, a *P* value < *α* implies a low probability that the *k* HLJDT's targets appear in the pathway by chance; that is, this pathway can be regarded as significantly regulated by HLJDT.

### 2.4. Network Scoring of Antirheumatic Effects of Drugs

#### 2.4.1. Scoring Network Effect of a Group of Seed Nodes

We applied the algorithm of random walk with restart to score the effect of a group of seed nodes on all the nodes in the network under study [38, 39]. The network is the weighted human PPI network, while the seeds could be disease-associated genes or protein targets of drugs.

A random walk starts at one of the seed nodes in the set *S*. At each step, the random walker either moves to a randomly chosen neighbor *u* ∈ *N* of the current node *v* or it restarts at one of the nodes in the seed set *S*. The probability of restarting at a given time step is a fixed parameter denoted by *r*. For each restart, the probability of restarting at *v* ∈ *S* suggests the degree of association between *v* and the seed set *S*. For each move, the probability of moving to interacting partner *u* of the current node *v* is proportional to the reliability of the interaction between *u* and *v*. After a sufficiently long time, the probability of being at node *v *at a random time step provides a measure of the functional association between *v* and the genes in seed set *S*. This process could be denoted as follows:

(3) $$\begin{array}{c} x^{t+1}=\left(1-r\right)Px^{t}+rx^{0}, \end{array}$$

where* P* is the adjacency matrix of the weighted PPI network, representing the coupling strength of nodes in the network; *r* ∈ [0,1] is a parameter denoting the restart probability which needs to be calibrated with real data; *x* ^*t*^ is a vector in which *x* ^*t*^(*v*) denotes the probability that the random walker will be at node *v* at time *t*; *x* ^0^ is a vector corresponding to the strength of seed nodes. The effect strength of seed set *S* to each nodes in the network is defined by steady-state probability vector *x* ^*∞*^ when *x* ^*t*+1^ = *x* ^*t*^.

The algorithm of random walk with start has been successfully used in the prioritization of candidate disease genes and *r* = 0.3 appeared to be a robust choice [40]. Thus we took *r* = 0.3 in this study.

#### 2.4.2. Scoring RA's Effect on the Human PPI Network

In this case the seed nodes are defined as RA-associated genes we collected. Theoretically, the degree in which different RA-associated gene correlates with RA is varying, and thus the initial strength values of different seed nodes should be different. For simplicity, we treated all RA-associated genes equally and defined the initial vector **x** ^0^ as *x* ^0^(*v*) = 1 if *v* is a seed; otherwise, *x* ^0^(*v*) = 0.

Then random walk with restart was used to compute the RA effect score of each node in the human network and we get a disease effect vector **x**_RA_.

#### 2.4.3. Scoring a Drug's Effect on the Human PPI Network

In this case, the seed nodes are defined as the drug's protein targets and the initial strength value of a seed node should be the binding strength or affinity of the drug to the corresponding target. In theory, the affinities could be measured in biochemical assays, which are not always available. Some studies used chemical proteomics data as a proxy for binding strengths [41, 42]. Here we study HLJDT's effect on the human PPI network by comparison with those of FDA-approved anti-RA drugs; thus, our focus is on the relative binding affinities of western drugs and HLJDT's components to target proteins. It has been known that the inhibition potency of natural compounds on protein targets is usually much lower than that of specifically designed drug molecules; for example, our earlier study found that the IC50 value of natural compound Astragaloside IV against proteins CN and ACE was approximately two orders higher than the corresponding western drugs cyclosporine A and enalapril, respectively [43]. Therefore, for an FDA-approved anti-RA drug, we defined the initial vector **x** ^0^ as *x* ^0^(*v*) = 1 if *v* is a seed; otherwise, *x* ^0^(*v*) = 0. Meanwhile, we defined the initial vector **x** ^0^ of a HLJDT's component as *x* ^0^(*v*) = 0.01 if *v* is a target of this component; otherwise, *x* ^0^(*v*) = 0.

For each drug, random walk with restart was used to compute its effect score on each node in the human network and we get its drug effect vector **x**_drug_.

#### 2.4.4. Scoring the Antirheumatic Effects of a Drug

We applied the inner product between the vectors of disease effect and drug effect to measure how the drug impacts the human interactome under the influence of the disease [42]. *E* = 〈*x*_RA_, *x*_drugk_〉 is defined specifically as the antirheumatic effect score of the *k*th drug under study. The effect score of a drug was then compared with that of its random contracts by *z*-score.

### 2.5. *Z*-Score

*Z*-score was applied to quantify the difference between the antirheumatic effect scores of a drug and its random counterparts as

(4) $$\begin{array}{c} z=\frac{E-{\overset{-}{E}}_{r}}{\Delta E_{r}}, \end{array}$$

where *E* is the score of antirheumatic effect of a drug and $\overset{-}{E}$ and Δ*E*_*r*_ are the mean and standard deviation of the corresponding metric for the random counterparts. The higher the absolute value of a *z*-score, the more significant the difference.

### 2.6. Construction of RA-Associated PPI Network

We defined RA-associated PPI network as a subnetwork of human PPI network consisting of nodes with high RA effect score. We sorted RA's effect scores and collected the top 3% proteins. Then these proteins and their interactions were extracted from human PPI network to construct the RA-associated PPI network.

### 2.7. Topological Features of Nodes in RA-Associated PPI Network

*Node Degree*. The degree of a node in a network is the number of connections it has to other nodes.

*k-Core*. A *k*-core of a graph is a maximal connected subgraph in which every vertex is connected to at least *k* vertices in the subgraph [44]. A *k*-core subgraph of a graph can be generated by recursively deleting the vertices from the graph whose present degree is less than *k*. This process can be iterated to gradually zoom into the more connected parts of the network. A node located in higher-level core indicates its higher centrality in the network.

*Betweenness Centrality*. Betweenness centrality is a measure of a node's centrality in a network [45]. It is equal to the number of the shortest paths from all vertices to all others that pass through that node. Betweenness centrality is a more useful measure (than just connectivity) of both the load and importance of a node. The betweenness centrality of a node *v* is given by the following equation:

(5) $$\begin{array}{c} g\left(v\right)=\sum_{s\neq v\neq t}\frac{\sigma_{st}\left(v\right)}{\sigma_{st}}, \end{array}$$

where *σ*_*st*_ is the total number of shortest paths from node *s* to node *t* and *σ*_*st*_(*v*) is the number of those paths that pass through node *v*.

## 3. Results and Discussion

### 3.1. HLJDT's Targets in the Drug-Target Network for Anti-RA Drugs

**Figure 1:**
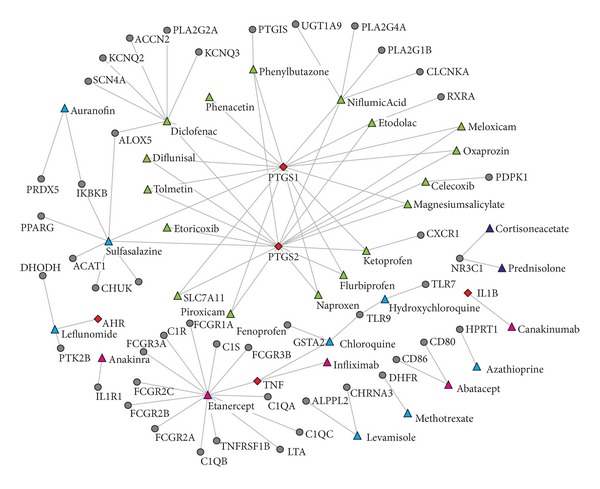
Drug-target network for all FDA approved anti-RA drugs in DrugBank. A target protein node and a drug node are linked if the protein is targeted by the corresponding drug. Triangles are drugs, while circles and diamonds are targets. Green: Nonsteroidal anti-inflammatory drugs; Shallow blue: Disease-modifying anti-rheumatic drugs; Dark blue: Glucocorticoids; Pink: Biological response modifiers; Red: Overlapped drug targets of FDA approved anti-RA drugs and HLJDT.

It would be interesting to bridge HLJDT and existing FDA-approved anti-RA drugs via their common drug targets. This is expected to provide alternative insights for deducing the therapeutic mechanism of HLJDT. We constructed the drug-target network for the 32 FDA-approved anti-RA drugs included in DrugBank and their corresponding 51 targets and then mapped the 91 targets of HLJDT onto this network. As shown in Figure 1, this network shows that the active compounds of HLJDT share 5 targets (TNF, PTGS1, PTGS2, AHR, and IL1B) with 3 types of anti-RA drugs, in which PTGS1, PTGS2, and TNF are conformed therapeutic targets for nonsteroidal anti-inflammatory drugs (NSAID) and biological response modifiers, respectively, suggesting that the effect of HLJDT could be a combination of different classes of anti-RA agents.

On the other hand, ZHENG is the key pathological principle in the TCM theory to understand disease pathogenesis and guide the treatment, in which the “Cold” ZHENG and “Hot” ZHENG are the two key statuses which therapeutically direct the use of TCM recipe in the clinical practice. It has been found that two targets of HLJDT, TNF, and IL1B are main hub nodes in the Hot ZENG network, implying the key roles that these proteins play in diseases related to Hot ZENG [46]. Therefore, from TCM theory, HLJDT as a hot-cooling TCM formula clears “heat” and “poison” by targeting the hub nodes of Hot ZENG network.

### 3.2. Pathways Significantly Regulated by HLJDT

RA is a systemic autoimmune disease which causes recruitment and activation of inflammatory cells, synovial hyperplasia, and destruction of cartilage and bone. The course of RA is accompanied with the prolonged and enhanced activation of the immune system, leading to the disturbance of the balance between bone formation and bone resorption, which results in periarticular bone destruction. Multiple inflammatory signaling pathways such as cytokine pathway and Wnt signaling are known to strigger the generation of bone resorbing osteoclasts [47].

To deduce the possible pathways affected by HLJDT, we mapped HLJDT's targets onto KEGG pathways of basic biological process, including pathways in metabolism, organismal systems, cellular processes, environmental information processing, and genetic information processing. A pathway enrichment analysis was performed to identify the pathways significantly affected by HLJDT, and *P* values were computed for each of the pathways with HLJDT's targets. Considering that diseases are higher level biological processes caused by the dysfunctions of basic biological processes, we did not include the KEGG pathway section of human diseases in this statistical analysis. The computation generated 32 pathways with values of *P* < 0.01, which may be regarded as key pathways affected by HLJDT (see Supplementary Table S4). In Table 1, we listed the 13 most significantly affected pathways with *P* value <10^−4^.

A central feature of RA is inflammation, one of the first responses of the immune system to infection or irritation. As listed in Table 1 and Supplementary Table S4, HLJDT acts on a large fraction of pathways in immune system. Some other pathways, although not classified into immune system in the KEGG database, have been known to be highly associated with the function of immune response, such as apoptosis [48] and MAPK signaling pathway [49]. Table 1 includes specifically several pathways related to pathogen recognition and inflammatory signalling in innate immune defences, in which the most important one is the Toll-like receptor (TLR) signalling pathway. The innate immune system relies on pattern recognition receptors (PRRs) to detect distinct pathogen-associated molecular patterns (PAMPs). Upon PAMP recognition, PRRs trigger a number of different signal transduction pathways. The pathways induced by PRRs ultimately result in the expression of a variety of proinflammatory molecules, such as cytokines, chemokines, cell-adhesion molecules, and immunoreceptors, which together orchestrate the early host response to infection, mediate the inflammatory response, and also bridge the adaptive immune response together [50]. The family of TLRs is the major class of PRRs [50]. In addition, we also found that HLJDT regulates some proinflammatory molecule-involved pathways, such as the chemokine signaling pathway, natural killer-cell mediated cytotoxicity, and Fc epsilon RI signaling pathway. These pathways indicate the process of innate immune response in the progress of RA. On the other hand, it is known that B and T lymphocytes are responsible for the adaptive immune response [51]. Table 1 shows that HLJDT's targets are involved in B- and T-cell receptor signalling pathways, implying that they regulate the adaptive immune response of RA.

Another prominent feature of RA is enhanced osteoclast formation, which disturbs the balance between bone resorption and bone formation. The osteoclast differentiation pathway is a biological process that maintains bone density and structure through a balance of bone resorption by osteoclasts and bone deposition by osteoblasts, while the WNT pathway regulates the balance between osteoclast and osteoblast function [52]. As can be seen in Table 1 and Supplementary Table S4, HLJDT's targets are significantly enriched in these two pathways, suggesting its function in tuning the imbalanced status.

Table 1 also tells us that HLJDT acts on the cytokine-cytokine receptor interaction pathway. An earlier study has found that immune factors are predominant in the Hot ZHENG network, and genes related to Hot ZHENG-related diseases are mainly present in the cytokine-cytokine receptor interaction pathway [46]. Thus from the perspective of TCM theory, HLJDT performs its therapeutic function by acting on the Hot ZENG network.

**Figure 2:**
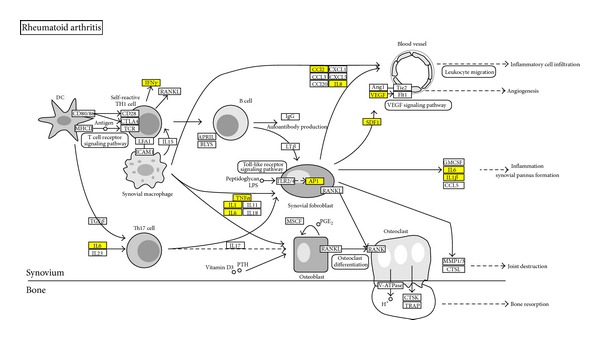
Regulations of HLJDT's active compounds on different proteins on RA pathway. Yellow boxes represent targets of HLJDT's active compounds. The original pathway map was downloaded from the KEGG database.

To see how HLJDT acts on the biological processes of RA, we then mapped the targets of HLJDT on the RA pathway in the KEGG database [31]. It was found that 12 of the 91 targets appear on this pathway (Figure 2). Figure 2 shows that HLJDT intervenes in the RA pathway by inhibiting multiple cytokines localized at its three distinct but associated developing branches of the disease, thus retarding the processes of inflammatory cell infiltration, inflammatory synovial pannus formation, and joint destruction. This suggests the therapeutic effect of HLJDT on RA.

### 3.3. Antirheumatic Effects of HLJDT Compared with Those of FDA-Approved Drugs by Network Scores

To quantitatively compare the antirheumatic effect of HLJDT with those of FDA-approved anti-RA drugs, we chose several representatives from each of the four classes of anti-RA western medicines and then computed the network score for the antirheumatic effect of each drug, respectively. The initial vector *x* ^0^ of drug effect was defined as *x* ^0^(*v*) = 1 if node *v* is a drug target; otherwise, *x* ^0^(*v*) = 0.

As shown in Table 2, biological response modifiers and disease-modifying antirheumatic drugs (DMARDs) get averagely much higher scores than the other two classes of drugs, nonsteroidal, anti-inflammatory drugs (NSAID) and glucocorticoids. Actually, biological response modifiers are a new type of DMARDs [53], that is, biotech agents, while drugs categorized into the class of DMARDs are small molecular compounds. DMARDs target the part of the immune system that is leading to inflammation and joint damage. Thus they can often slow or stop the progression of RA. From Table 2, we can see that some DMARDs target directly on RA-associated genes such as TNF, CD80, and CD86 [54], supporting their higher antirheumatic effects.

Since RA is an inflammatory disease affecting the joints, it gets worse over time unless the inflammation is stopped or slowed. Thus anti-inflammatory is very important in the treatment. Glucocorticoids and NSAIDs are such class of drugs, in which glucocorticoids are steroidal strong anti-inflammatory drugs that can also block other immune responses while NSAIDs work by inhibiting enzymes that promote inflammation [55]. By reducing inflammation, anti-inflammatory agents help reduce swelling and pain. But they are not effective in reducing joint damage. Thus these drugs alone are not effective in treating the disease and they should be taken in combination with other rheumatoid arthritis medications [56].

We then computed the network score for the antirheumatic effect of HLJDT and its compounds, respectively. Unlike specifically designed drug molecules, HLJDT's active compounds are naturally occurring substances; thus, their inhibition potency on targets could be much weaker. Therefore, we defined the initial vector *x* ^0^ of HLJDT's components as *x* ^0^(*v*) = 0.01 if node *v* is a target; otherwise, *x* ^0^(*v*) = 0. In this way, the antirheumatic effect score of HLJDT and its compounds are obtained as listed in Table 3. It can be seen that the effect score of each component is very small, while the whole HLJDT achieves a much higher effect score, which is in the same order as that of anti-inflammatory agents, including glucocorticoids and NSAIDs. This result quantitatively validates the suggestion that weak inhibition of multiple targets could orchestrate synergistic effect comparable to strong inhibition of a single target [57].

To investigate if the scores of HLJDT and its components suggest significant antirheumatic effect, for each drug, we generated 3000 random target sets, respectively, each of which included the same number of proteins as the drug's targets. The mean effect score and the standard deviation of the 3000 random counterparts were calculated. Hence the *z*-score of HLJDT and its compounds' antirheumatic effect score were obtained, which were listed in Table 3. The absolute value of *z*-score bigger than 3 usually suggests a statistically significant deviation between the actual value and the random ones. Thus the *z*-score 21.12 of HLJDT suggests its significant antirheumatic effect. The *z*-scores of four active compounds, berberine, coptisine, wogonin, and baicalein, are greater than 3.0, implying the antirheumatic effect of these single compounds. In fact, an earlier study has reported the effects of these compounds on RA [15–18].

### 3.4. HLJDT's Effects on RA-Associated PPI Network

**Figure 3:**
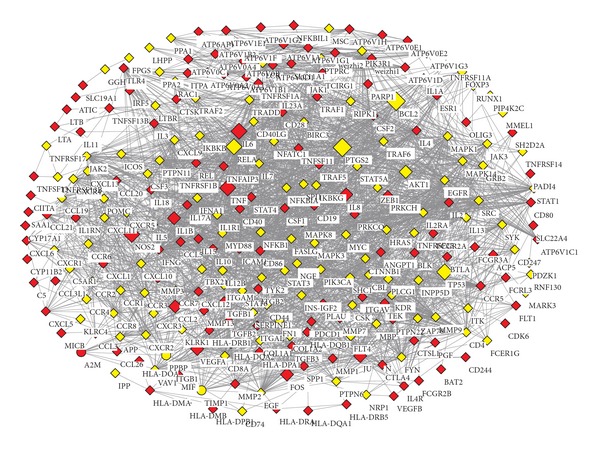
HLJDT's effects on RA-associated PPI network. This network consists of proteins with high RA effect score and their interactions. Diamond nodes are overlapped target proteins of HLJDT, while the size of a diamond node corresponds to the number of HLJDT's components targeting on this protein. Red: RA-associated genes; Yellow: other genes.

To see how HLJDT acts on a protein-protein interaction network affected by RA, we first constructed an RA-associated PPI network, which consists of proteins with top 3% RA effect scores and their interactions. This network has 272 nodes and 2803 edges. Of the 163 RA-associated genes under study, 151 ones appear on this network, taking a percentage of 93.79%, suggesting a high correlation of this network to RA's biological process. Then the 91 target proteins of HLJDT were mapped onto this RA-associated PPI network and 28 of which were found on this network, in which half are targeted by multiple components of HLJDT. As shown in Figure 3, HLJDT acts on 12 RA-associated genes, while some major causative genes of RA in this network, such as TNF and ILs are targeted by HLJDT's multiple components.

To understand the roles that HLJDT's targets play on the RA-associated PPI network, we analyzed three topological features which reflect node centrality in this network, including degree, betweenness, and *k*-core. The average degree and betweenness of nodes in this network are 20 and 0.0063, respectively, and the highest *k*-core index is 20. In Table 4 we listed the three topological measures of the 28 HLJDT's targets in this network. It can be seen that most targets located in the highest *k*-core and have degrees higher than the average, and the betweenness values of more than half targets are higher than the average, suggesting that HLJDT may interfere with RA by acting on proteins in the central locations of the disease network with multiple components.

## 4. Conclusions

This work studies HLJDT's antirheumatic effects from a network perspective. We have extracted data related to RA's pathogenesis and treatment—RA-associated genes from the OMIM database, GAD and KEGG pathway database, protein targets of FDA-approved anti-RA drugs, and HLJDT, respectively. First, we constructed drug-target network for FDA-approved anti-RA drugs. By mapping HLJDT's targets on this network, we found that 5 targets of HLJDT, TNF, PTGS1, PTGS2, AHR, and IL1B, exist in this network. Then we mapped HLJDT's targets onto KEGG pathways of basic biological process and identified 32 pathways enriched with HLJDT's targets, which include pathways in immune system and bone formation. These pathways are considered as key pathways affected by HLJDT. In addition, 12 targets were found involved in the KEGG RA pathway. These findings indicate that HLJDT could intervene in the biological process of the occurrence and development of RA by targeting on multiple targets associated with immune function and bone modeling, and it may function as a combination of different categories of anti-RA drugs.

We also quantitatively analyzed the antirheumatic effect of HLJDT and compared it with those of FDA-approved anti-RA drugs through a network based antirheumatic effect score. It is found that the antirheumatic effect score of each HLJDT's component is very low, while the whole HLJDT achieves a much higher effect score, which is comparable to that of FDA approved anti-inflammatory agents. This result suggests a synergistic antirheumatic effect of HLJDT achieved by its multiple components acting on multiple targets.

At last, we conducted topological analysis on the RA-associated PPI network to investigate the roles HLJDT's targets play on this network. We found that most targets own large degree, betweenness, and high *k*-core index in the network, suggesting that HLJDT may interfere with RA by acting on proteins in the central locations of the disease network with multiple components.

In TCM theory, RA could be related to Cold ZHENG or Hot ZHENG [58]. Our study on drug-target network and pathways also found that HLJDT targets on hub nodes and main pathway in the Hot ZENG network, suggesting that HLJDT could be applied as adjuvant treatment for Hot-ZENG-related RA. Further clinical trial needs to be conducted to confirm this.

This work applied network approach to explain HLJDT's antirheumatic effect. It may shed new lights on the study about the TCM pharmacology and promote the development of nationality medicine.

## Supplementary Material

Table S1: Genes associated with RA collected from three resources: OMIM, GAD and KEGG.
Table S2: FDA approved anti-RA drugs and their target proteins collected from GrugBank.
Table S3: Target proteins of HLJDT's main ingredients collected from HIT.
Table S4: Pathways significantly regulated by HLJDT.

## Figures and Tables

**Table 1:** KEGG pathways significantly enriched with targets of HLJDT's components.

Pathway class	Pathway name	Total genes on pathway	HLJDT's targets on pathway
Cell communication	Focal adhesion	200	14
Cell growth and death	p53 signaling pathway	69	13
	Apoptosis	86	14
Development	Osteoclast differentiation	128	13
Immune system	Toll-like receptor signaling pathway	102	13
	T-cell receptor signaling pathway	108	13
	NOD-like receptor signaling pathway	59	9
	B-cell receptor signaling pathway	75	9
	Chemokine signaling pathway	189	11
Nervous system	Neurotrophin signaling pathway	127	12
Signal transduction	VEGF signaling pathway	76	10
	MAPK signaling pathway	272	15
Signaling molecules and interaction	Cytokine-cytokine receptor interaction	275	15

**Table 2:** The anti-rheumatic effect scores of representative anti-RA western medicines.

Class of drug	Anti-RA drug	Targets	Effect score
Biological response modifiers	Etanercept	FCGR2C, **TNFRSF1B**, **TNF**, **LTA**, FCGR3B, **FCGR3A**, **FCGR2B**, **FCGR2A**, FCGR1A, C1S, C1R, C1QC, C1QB, C1QA	1.644
	Abatacept	**CD86**, **CD80**	0.609
	Infliximab	**TNF**	0.293
	Anakinra	**IL1R1**	0.159

DMARDs	Chloroquine	TLR9, **TNF**, GSTA2	0.463
	Sulfasalazine	SLC7A11, **PTGS2**, PTGS1, PPARG, **IKBKB**, CHUK, ALOX5, ACAT1	0.454
	Hydroxychloroquine	TLR9, TLR7	0.173
	Leflunomide	PTK2B, DHODH, AHR	0.149
	Auranofin	PRDX5, **IKBKB**	0.14
	Leflunomide	PTK2B, DHODH, AHR	0.061
	Azathioprine	HPRT1	0.053
	Auranofin	PRDX5, **IKBKB**	0.05

NSAIAs	Flurbiprofen	**PTGS2**, PTGS1	0.133

Glucocorticoids	Cortisone acetate	NR3C1	0.063

RA-associated disease genes are marked in bold characters.

**Table 3:** The anti-rheumatic effect scores of HLJDT and its main ingredients.

The component of HLJDT	Target numbers	Effect Score	*Z*-score
HLJDT	78	0.137	21.122
Berberine	52	0.061	9.635
Coptisine	6	0.0139	7.827
Wogonin	26	0.032	7.627
Baicalein	24	0.0215	4.377
Chlorogenic	1	0.001	1.914
Crocetin	1	0.001	1.457
Geniposide	5	0.002	0.468
Palmatine	1	0.0003	−0.011
Jatrorrhizine	1	0.0002	−0.260

**Table 4:** The network topology analysis about the overlapped genes and target proteins of HLJDT. It mainly included degree of distribution, betweenness, and K-core analysis.

Gene	Degree	Betweenness	K-coreness	RA disease gene	Targeted by component of HLJDT
IL6	84	0.059	20	Y	Berberine; coptisine; wogonin
IFNG	75	0.047	20	Y	Berberine
IL1B	63	0.029	20	Y	Berberine; coptisine
JUN	59	0.016	20	Y	Berberine; wogonin
IL8	58	0.026	20	Y	Berberine; wogonin
VEGFA	51	0.027	20	Y	Baicalein; berberine
FOS	51	0.024	20	Y	Baicalein; berberine
IL4	50	0.014	20	Y	Berberine
CCL2	41	0.006	20	Y	Berberine; wogonin
CXCL12	33	0.009	19	Y	Berberine
NOS2	9	1.35 × 10^−5^	9	Y	Berberine; coptisine; wogonin
TNF	6	4.45 × 10^−5^	6	Y	Berberine; coptisine; wogonin
RELA	56	0.012	20	N	Baicalein; berberine; wogonin
SRC	48	0.026	20	N	Baicalein
IL2RA	44	0.003	20	N	Berberine
MAPK1	42	0.016	20	N	Berberine
NFKBIA	42	0.008	20	N	Berberine
AKT1	42	0.010	20	N	Baicalein; wogonin
MMP9	38	0.0130	20	N	Baicalein
EGFR	35	0.004	20	N	Berberine
FN1	34	0.010	15	N	Wogonin
BCL2	33	0.004	20	N	Baicalein; berberine; geniposide; wogonin
PTGS2	30	0.004	20	N	Baicalein; berberine; coptisine; wogonin
RAC1	26	0.002	19	N	Berberine
APP	24	0.006	16	N	Berberine
TP53	24	0.007	18	N	Baicalein; berberine; wogonin
KDR	22	0.003	15	N	Wogonin
NFATC1	20	0.003	18	N	Baicalein
