# Supplementary material for: Bioinformatics Analysis for the Antirheumatic Effects of Huang-Lian-Jie-Du-Tang from a Network Perspective
Source: Evid Based Complement Alternat Med. 2013 Nov 21;2013:245357. doi: 10.1155/2013/245357 (PMC3856148; doi:10.1155/2013/245357)
Supplement: Supplementary file 1 — Table S1: Genes associated with RA collected from three resources: OMIM, GAD and KEGG. Table S2: FDA approved anti-RA drugs and their target proteins collected from GrugBank. Table S3: Target proteins of HLJDT's main ingredients collected from HIT. Table S4: Pathways significantly regulated by HLJDT. [file 245357.f1.pdf]

Supplementary file for “Bioinformatics analysis for the anti-rheumatic effects of Huang-Lian-Jie-Du-Tang from a network perspective”

Contents

Table S1. Genes associated with RA from three resources
Table S2. FDA approved anti-RA drugs and their target proteins
Table S3. Target proteins of HLJDT’s main ingredients
Table S4. Pathways significantly regulated by HLJDT

Table S1 Genes associated with RA from three resources

Gene name	ID	Database
A2M	2	GAD
NAT2	10	GAD
ACP5	54	KEGG PASSWAY
PARP1	142	GAD
AMPD1	270	GAD
ANGPT1	284	KEGG PASSWAY
ATIC	471	GAD
ATP6V1A	523	KEGG PASSWAY
ATP6V1B1	525	KEGG PASSWAY
ATP6V1B2	526	KEGG PASSWAY
ATP6V0C	527	KEGG PASSWAY
ATP6V1C1	528	KEGG PASSWAY
ATP6V1E1	529	KEGG PASSWAY
ATP6V0B	533	KEGG PASSWAY
ATP6V1G2	534	KEGG PASSWAY
ATP6V0A1	535	KEGG PASSWAY
ATP6AP1	537	KEGG PASSWAY
BLK	640	GAD
C5	727	GAD
CD19	930	GAD
CD28	940	KEGG PASSWAY
CD80	941	KEGG PASSWAY
CD86	942	KEGG PASSWAY
CD40	958	GAD
CDK6	1021	GAD
CCR5	1234	GAD
CSF1	1435	KEGG PASSWAY
CSF2	1437	KEGG PASSWAY

CTLA4	1493	GAD;KEGG PASSWAY
CTSK	1513	KEGG PASSWAY
CTSL1	1514	KEGG PASSWAY
CYP11B2	1585	GAD
CYP17A1	1586	GAD
ESR1	2099	GAD
FCGR2A	2212	GAD
FCGR2B	2213	GAD
FCGR3A	2214	GAD
FLT1	2321	KEGG PASSWAY
FOS	2353	KEGG PASSWAY
CXCL1	2919	KEGG PASSWAY
HLA-DMA	3108	GAD;KEGG PASSWAY
HLA-DMB	3109	KEGG PASSWAY
HLA-DOA	3111	KEGG PASSWAY
HLA-DOB	3112	KEGG PASSWAY
HLA-DPA1	3113	KEGG PASSWAY
HLA-DPB1	3115	KEGG PASSWAY
HLA-DQA1	3117	GAD;KEGG PASSWAY
HLA-DQA2	3118	KEGG PASSWAY
HLA-DQB1	3119	GAD;KEGG PASSWAY
HLA-DRA	3122	KEGG PASSWAY
HLA-DR1B, SS1	3123	OMIM;GAD;KEGG PASSWAY
HLA-DRB3	3125	KEGG PASSWAY
HLA-DRB4	3126	KEGG PASSWAY
HLA-DRB5	3127	KEGG PASSWAY
ICAM1	3383	KEGG PASSWAY
IFNG	3458	GAD;KEGG PASSWAY
IL1A	3552	KEGG PASSWAY
IL1B	3553	GAD;KEGG PASSWAY
IL1RN	3557	GAD
IL2	3558	GAD
IL3	3562	GAD
IL4	3565	GAD
IL4R	3566	GAD
IL6	3569	GAD;KEGG PASSWAY
IL8	3576	KEGG PASSWAY
IL10	3586	GAD
IL11	3589	KEGG PASSWAY
IL15	3600	KEGG PASSWAY
IL17A	3605	KEGG PASSWAY
IL18	3606	GAD;KEGG PASSWAY
IRF5	3663	GAD

ITGAL	3683	KEGG PASSWAY
ITGAV	3685	GAD
ITGB2	3689	KEGG PASSWAY
ITPA	3704	GAD
JUN	3725	KEGG PASSWAY
KIR2DS2	3807	GAD
LTA	4049	GAD
LTB	4050	KEGG PASSWAY
MHC2TA, C2TA	4261	OMIM
MICA	4276	GAD
MICB	4277	GAD
MIF	4282	GAD
MMP1	4312	GAD;KEGG PASSWAY
MMP2	4313	GAD
MMP3	4314	GAD;KEGG PASSWAY
MMP7	4316	GAD
MMP12	4321	GAD
MMP13	4322	GAD
MTHFR	4524	GAD
NFKBIL1	4795	OMIM
NOS2A	4843	GAD
PDCD1	5133	GAD
PGF	5228	KEGG PASSWAY
PLAU	5328	GAD
PRKCH	5583	GAD
PRKCQ	5588	GAD
REL	5966	GAD
SAA1	6288	GAD
CCL2	6347	KEGG PASSWAY
CCL3	6348	KEGG PASSWAY
CCL3L1	6349	KEGG PASSWAY
CCL5	6352	KEGG PASSWAY
CCL20	6364	KEGG PASSWAY
CCL21	6366	GAD
CXCL6	6372	KEGG PASSWAY
CXCL5	6374	KEGG PASSWAY
CXCL12	6387	KEGG PASSWAY
SLC11A1	6556	GAD
SLC19A1	6573	GAD
SLC22A4, OCTN1	6583	OMIM;GAD
SPP1	6696	GAD
STAT4	6775	GAD
TAP2	6891	GAD

TRB@	6957	GAD
TEK	7010	KEGG PASSWAY
TGFB1	7040	KEGG PASSWAY
TGFB2	7042	KEGG PASSWAY
TGFB3	7043	KEGG PASSWAY
TLR2	7097	KEGG PASSWAY
TLR4	7099	KEGG PASSWAY
TNF	7124	GAD;KEGG PASSWAY
TNFAIP3	7128	GAD
TNFRSF1A	7132	GAD
TNFRSF1B	7133	GAD
TRAF1	7185	GAD
TRAF5	7188	GAD
VEGFA	7422	GAD;KEGG PASSWAY
VEGFB	7423	KEGG PASSWAY
BAT2	7916	GAD
TNFSF11	8600	KEGG PASSWAY
TNFSF13	8741	KEGG PASSWAY
TNFRSF11A	8792	KEGG PASSWAY
TIMELESS	8914	GAD
ATP6V0E1	8992	KEGG PASSWAY
SH2D2A	9047	GAD
ATP6V0D1	9114	KEGG PASSWAY
ATP6V1F	9296	KEGG PASSWAY
ATP6V1G1	9550	KEGG PASSWAY
TCIRG1	10312	KEGG PASSWAY
CCL26	10344	GAD
TNFSF13B	10673	KEGG PASSWAY
ATP6V0A2	23545	KEGG PASSWAY
PADI4, PADI5, PAD	23569	OMIM;GAD
PTPN22, PEP, PTPN8, LYP	26191	OMIM;GAD
SALL3	27164	GAD
IGLV8	28774	GAD
ATP6V0A4	50617	KEGG PASSWAY
ATP6V1D	51382	KEGG PASSWAY
IL23A	51561	KEGG PASSWAY
ATP6V1H	51606	KEGG PASSWAY
CD244, NAIL, NKR2B4, SLAMF4	51744	OMIM
EXOC4	60412	GAD
ATP6V1E2	90423	KEGG PASSWAY
FCRL3	115352	GAD
ATP6V1G3	127124	KEGG PASSWAY
CCBE1	147372	GAD

BTLA	151888	GAD
ATP6V0E2	155066	KEGG PASSWAY
OLIG3	167826	GAD
CCL3L3	414062	KEGG PASSWAY

Table S2. FDA approved anti-RA drugs and their target proteins

DRUG TYPE	DRUG NAME	DRUG TARGET	TARGET ID	TARGET MEANS
Biotech Agents	Anakinra	IL1R1	3554	Interleukin-1 receptor type I
	Infliximab	TNF	7124	Tumor necrosis factor
	Abatacept	CD86	942	T-lymphocyte activation antigen CD86
		CD80	941	T-lymphocyte activation antigen CD80
	Etanercept	FCGR2C	9103	Low affinity immunoglobulin gamma Fc region receptor II-c
		TNFRSF1B	7133	Tumor necrosis factor receptor superfamily member 1B
		TNF	7124	Tumor necrosis factor
		LTA	4049	Lymphotoxin-alpha
		FCGR3B	2215	Low affinity immunoglobulin gamma Fc region receptor III-B
		FCGR3A	2214	Low affinity immunoglobulin gamma Fc region receptor III-A
		FCGR2B	2213	Low affinity immunoglobulin gamma Fc region receptor II-b
		FCGR2A	2212	Low affinity immunoglobulin gamma Fc region receptor II-a
		FCGR1A	2209	High affinity immunoglobulin gamma Fc receptor I
		C1S	716	Complement C1s subcomponent
		C1R	715	Complement C1r subcomponent
		C1QC	714	Complement C1q subcomponent subunit C
		C1QB	713	Complement C1q subcomponent subunit B
		C1QA	712	Complement C1q subcomponent subunit A
	Canakinumab	IL1B	3553	Interleukin-1 beta
NSAIAs	Celecoxib	PTGS2	5743	Prostaglandin G/H synthase 2
		PDPK1	5170	3-phosphoinositide-dependent protein kinase 1
	Piroxicam	PTGS2	5743	Prostaglandin G/H synthase 2
		PTGS1	5742	Prostaglandin G/H synthase 1
	Etodolac	RXRA	6256	Retinoic acid receptor RXR-alpha
		PTGS2	5743	Prostaglandin G/H synthase 2
		PTGS1	5742	Prostaglandin G/H synthase 1
	Etoricoxib	PTGS2	5743	Prostaglandin G/H synthase 2

	Tolmetin	PTGS2	5743	Prostaglandin G/H synthase 2
		PTGS1	5742	Prostaglandin G/H synthase 1
	Fenoprofen	PTGS2	5743	Prostaglandin G/H synthase 2
		PTGS1	5742	Prostaglandin G/H synthase 1
	Diclofenac	SCN4A	6329	Sodium channel protein type 4 subunit alpha
		PTGS2	5743	Prostaglandin G/H synthase 2
		PTGS1	5742	Prostaglandin G/H synthase 1
		PLA2G2A	5320	Phospholipase A2, membrane associated
		KCNQ3	3786	Potassium voltage-gated channel subfamily KQT member 3
		KCNQ2	3785	Potassium voltage-gated channel subfamily KQT member 2
		ALOX5	240	Arachidonate 5-lipoxygenase
		ACCN2	41	Amiloride-sensitive cation channel 2, neuronal
	Flurbiprofen	PTGS2	5743	Prostaglandin G/H synthase 2
		PTGS1	5742	Prostaglandin G/H synthase 1
	Naproxen	PTGS2	5743	Prostaglandin G/H synthase 2
		PTGS1	5742	Prostaglandin G/H synthase 1
	Phenylbutazone	PTGS2	5743	Prostaglandin G/H synthase 2
		PTGS1	5742	Prostaglandin G/H synthase 1
		PTGIS	5740	Prostacyclin synthase
	Meloxicam	PTGS2	5743	Prostaglandin G/H synthase 2
		PTGS1	5742	Prostaglandin G/H synthase 1
	Diflunisal	PTGS2	5743	Prostaglandin G/H synthase 2
		PTGS1	5742	Prostaglandin G/H synthase 1
	Oxaprozin	PTGS2	5743	Prostaglandin G/H synthase 2
		PTGS1	5742	Prostaglandin G/H synthase 1
	NiflumicAcid	UGT1A9	54600	UDP-glucuronosyltransferase 1-9
		PTGS2	5743	Prostaglandin G/H synthase 2
		PTGS1	5742	Prostaglandin G/H synthase 1
		PLA2G4A	5321	Cytosolic phospholipase A2
		PLA2G1B	5319	Phospholipase A2
		CLCNKA	1187	Chloride channel protein ClC-Ka
	Ketoprofen	PTGS2	5743	Prostaglandin G/H synthase 2
		PTGS1	5742	Prostaglandin G/H synthase 1
		CXCR1	3577	High affinity interleukin-8 receptor A
	Phenacetin	PTGS1	5742	Prostaglandin G/H synthase 1
	Magnesiumsalicylate	PTGS2	5743	Prostaglandin G/H synthase 2
		PTGS1	5742	Prostaglandin G/H synthase 1
DMARDs	Chloroquine	TLR9	54106	Toll-like receptor 9
		TNF	7124	Tumor necrosis factor
		GSTA2	2939	Glutathione S-transferase A2
	Sulfasalazine	SLC7A11	23657	Cystine/glutamate transporter

		PTGS2	5743	Prostaglandin G/H synthase 2
		PTGS1	5742	Prostaglandin G/H synthase 1
		PPARG	5468	Peroxisome proliferator-activated receptor gamma
		IKBKB	3551	Inhibitor of nuclear factor kappa-B kinase subunit beta
		CHUK	1147	Inhibitor of nuclear factor kappa-B kinase subunit alpha
		ALOX5	240	Arachidonate 5-lipoxygenase
		ACAT1	38	Acetyl-CoA acetyltransferase, mitochondrial
	Levamisole	CHRNA3	1136	Neuronal acetylcholine receptor subunit alpha-3
		ALPPL2	251	Alkaline phosphatase, placental-like
	Azathioprine	HPRT1	3251	Hypoxanthine-guanine phosphoribosyltransferase
	Auranofin	PRDX5	25824	Peroxiredoxin-5, mitochondrial
		IKBKB	3551	Inhibitor of nuclear factor kappa-B kinase subunit beta
	Hydroxychloroquine	TLR9	54106	Toll-like receptor 9
		TLR7	51284	Toll-like receptor 7
	Leflunomide	PTK2B	2185	Protein tyrosine kinase 2 beta
		DHODH	1723	Dihydroorotate dehydrogenase, mitochondrial
		AHR	196	Aryl hydrocarbon receptor
	Methotrexate	DHFR	1719	Dihydrofolate reductase
Glucocorticoids	Prednisolone	NR3C1	2908	Glucocorticoid receptor
	Cortisoneacetate	NR3C1	2908	Glucocorticoid receptor

Table S3. Target proteins of HLJDT's main ingredients

Gene ID	Gene Name	Target Mean	Component
183	AGT	Angiotensinogen	berberine
196	AHR	Aryl hydrocarbon receptor	baicalein;berberine
207	AKT1	RAC-alpha serine/threonine-protein kinase	baicalein;wogonin
239	ALOX12	Arachidonate 12-lipoxygenase, 12S-type	baicalein
347	APOD	Apolipoprotein D	baicalein
351	APP	Amyloid beta A4 protein	berberine
581	BAX	Apoptosis regulator BAX	baicalein;berberine;wogonin
595	CCND1	G1/S-specific cyclin-D1	berberine;berberine;coptisine;wogonin
596	BCL2	Apoptosis regulator Bcl-2	baicalein;berberine;geniposide;wogonin

598	BCL2L1	Bcl-2-like protein 1	berberine
637	BID	BH3-interacting domain death agonist	berberine
836	CASP3	Caspase-3	baicalein;berberine;wogonin
841	CASP8	Caspase-8	berberine
842	CASP9	Caspase-9	berberine;wogonin
862	RUNX1T1	Protein CBFA2T1	berberine
891	CCNB1	G2/mitotic-specific cyclin-B1	baicalein;berberine
969	CD69	Early activation antigen CD69	berberine
998	CDC42	Cell division control protein 42 homolog	berberine
1017	CDK2	Cell division protein kinase 2	berberine
1019	CDK4	Cell division protein kinase 4	berberine
1026	CDKN1A	Cyclin-dependent kinase inhibitor 1	berberine;wogonin
1543	CYP1A1	Cytochrome P450 1A1	berberine
1649	DDIT3	DNA damage-inducible transcript 3 protein	berberine
1956	EGFR	Epidermal growth factor receptor	berberine
1958	EGR1	Early growth response protein 1	berberine
2171	FABP5	Fatty acid-binding protein, epidermal	baicalein
2335	FN1	Fibronectin	wogonin
2353	FOS	Proto-oncogene c-Fos	baicalein;berberine
2355	FOSL2	Fos-related antigen 2	baicalein
2641	GCG	Glucagon	geniposide
2694	GIF	Gastric intrinsic factor	chlorogenic acid
2932	GSK3B	Glycogen synthase kinase-3 beta	wogonin
2944	GSTM1	Glutathione S-transferase Mu 1	geniposide
2946	GSTM2	Glutathione S-transferase Mu 2	geniposide
3091	HIF1A	Hypoxia-inducible factor 1-alpha	baicalein;berberine
3162	HMOX1	Heme oxygenase 1	geniposide
3172	HNF4A	Hepatocyte nuclear factor 4-alpha	berberine
3458	IFNG	Interferon gamma	berberine
3481	IGF2	Insulin-like growth factor II	baicalein
3553	IL1B	Interleukin-1 beta	berberine;coptisine
3559	IL2RA	Interleukin-2 receptor subunit alpha	berberine
3565	IL4	Interleukin-4	berberine
3569	IL6	Interleukin-6	berberine;coptisine;wogonin
3576	IL8	Interleukin-8	berberine;wogonin
3630	INS	Insulin	berberine

3692	EIF6	Eukaryotic translation initiation factor 6	berberine;wogonin
3725	JUN	Transcription factor AP-1	berberine;wogonin
3791	KDR	Vascular endothelial growth factor receptor 2	wogonin
4128	MAOA	Amine oxidase [flavin-containing] A	berberine;coptisine;jatrorrhizine
4129	MAOB	Amine oxidase [flavin-containing] B	jatrorrhizine
4170	MCL1	Induced myeloid leukemia cell differentiation protein Mcl-1	wogonin
4318	MMP9	Matrix metalloproteinase-9	baicalein
4353	MPO	Myeloperoxidase	baicalein;berberine
4772	NFATC1	Nuclear factor of activated T-cells, cytoplasmic 1	baicalein
4792	NFKBIA	NF-kappa-B inhibitor alpha	berberine
4843	NOS2	Nitric oxide synthase, inducible	berberine;coptisine;wogonin
5154	PDGFA	Platelet-derived growth factor subunit A	berberine
5580	PRKCD	Protein kinase C delta type	wogonin
5594	MAPK1	Mitogen-activated protein kinase 1	berberine
5742	PTGS1	Prostaglandin G/H synthase 1	wogonin
5743	PTGS2	Prostaglandin G/H synthase 2	baicalein;berberine;coptisine;wogonin
5832	ALDH18A1	Delta-1-pyrroline-5-carboxylate synthase	berberine
5879	RAC1	Ras-related C3 botulinum toxin substrate 1	berberine
5924	RASGRF2	Ras-specific guanine nucleotide-releasing factor 2	berberine
5970	RELA	Transcription factor p65	baicalein;berberine;wogonin
6347	CCL2	C-C motif chemokine 2	berberine;wogonin
6387	CXCL12	Stromal cell-derived factor 1	berberine
6390	SDHB	Succinate dehydrogenase [ubiquinone] iron-sulfur subunit, mitochondrial	jatrorrhizine
6476	SI	Sucrase-isomaltase, intestinal	berberine
6513	SLC2A1	Solute carrier family 2, facilitated glucose transporter member 1	berberine
6610	SMPD2	Sphingomyelin phosphodiesterase 2	crocin
6714	SRC	Proto-oncogene tyrosine-protein kinase Src	baicalein
7011	TEP1	Telomerase protein component 1	berberine;wogonin
7054	TH	Tyrosine 3-monooxygenase	berberine;palmatine
7124	TNF	Tumor necrosis factor	berberine;coptisine;wogonin

7157	TP53	Cellular tumor antigen p53	baicalein;berberine;wogonin
7173	TPO	Thyroid peroxidase	chlorogenic acid
7412	VCAM 1	Vascular cell adhesion protein 1	crocetin
7422	VEGF A	Vascular endothelial growth factor A	baicalein;berberine
8061	FOSL1	Fos-related antigen 1	baicalein
8972	MGAM	Maltase-glucoamylase, intestinal	berberine
9429	ABCG 2	ATP-binding cassette sub-family G member 2	berberine
10598	AHSA1	Activator of 90 kDa heat shock protein ATPase homolog 1	wogonin
23424	TDRD7	Tudor domain-containing protein 7	baicalein
27113	BBC3	Bcl-2-binding component 3	wogonin
51083	GAL	Galanin	berberine
51191	HERC5	Probable E3 ubiquitin-protein ligase HERC5	berberine
54205	CYCS	Cytochrome c	baicalein;berberine
54583	EGLN1	Egl nine homolog 1	baicalein
79400	NOX5	NADPH oxidase 5	baicalein
255738	PCSK9	Proprotein convertase subtilisin/kexin type 9	berberine

Table S4. Pathways significantly regulsted by HLJDT

Pathway Class	Pathway Name	Pathway total genes	Pathway mapped genes	p-value
Amino Acid Metabolism	Tyrosine metabolism	41	4	0.0020591
	Arginine and proline metabolism	55	4	0.0060231
Cell Communication	Focal adhesion	200	14	2.89E-07
	Adherens junction	73	5	0.0027764
Cell Growth and Death	p53 signaling pathway	69	13	6.50E-12
	Apoptosis	86	14	1.94E-11
	Cell cycle	128	7	0.0015054
Development	Osteoclast differentiation	128	13	9.67E-09
	Axon guidance	130	6	0.0075546
Endocrine System	Adipocytokine signaling pathway	69	5	0.0021653
	GnRH signaling pathway	101	6	0.0021779
	Progesterone-mediated oocyte maturation	87	5	0.0059095
Immune System	Toll-like receptor signaling pathway	102	13	5.71E-10
	T cell receptor signaling pathway	108	13	1.17E-09
	NOD-like receptor signaling pathway	59	9	6.24E-08

	B cell receptor signaling pathway	75	9	5.25E-07
	Chemokine signaling pathway	189	11	3.59E-05
	Fc epsilon RI signaling pathway	79	6	0.0006025
	Hematopoietic cell lineage	88	6	0.0010682
	RIG-I-like receptor signaling pathway	71	5	0.0024571
	Natural killer cell mediated cytotoxicity	141	7	0.0026206
	Intestinal immune network for IgA production	49	4	0.0039744
	Fc gamma R-mediated phagocytosis	95	5	0.0085326
	Cytosolic DNA-sensing pathway	62	4	0.0091839
Nervous System	Neurotrophin signaling pathway	127	12	8.58E-08
Signal Transduction	VEGF signaling pathway	76	10	4.73E-08
	MAPK signaling pathway	272	15	2.22E-06
	ErbB signaling pathway	87	7	0.0001441
	mTOR signaling pathway	52	5	0.0005974
	Jak-STAT signaling pathway	155	8	0.0010035
	Wnt signaling pathway	151	7	0.0038453
Signaling Molecules and Interaction	Cytokine-cytokine receptor interaction	275	15	2.55E-06
